# Modulation of Gut Microbiota Through Dietary Intervention in Neuroinflammation and Alzheimer’s and Parkinson’s Diseases

**DOI:** 10.1007/s13668-024-00539-7

**Published:** 2024-04-23

**Authors:** Şerife Ayten, Saniye Bilici

**Affiliations:** Department of Nutrition and Dietetics, Gazi University, Ankara, Turkey

**Keywords:** Dietary intervention, Gut microbiota, Neurodegenerative diseases, Neuroinflammation

## Abstract

**Purpose of Review:**

The gut microbiota plays a crucial role in the pathogenesis of neuroinflammation and Alzheimer’s and Parkinson’s diseases. One of the main modulators of the gut microbiota is the diet, which directly influences host homeostasis and biological processes. Some dietary patterns can affect neurodegenerative diseases’ progression through gut microbiota composition, gut permeability, and the synthesis and secretion of microbial-derived neurotrophic factors and neurotransmitters. This comprehensive review critically assesses existing studies investigating the impact of dietary interventions on the modulation of the microbiota in relation to neurodegenerative diseases and neuroinflammation.

**Recent Findings:**

There are limited studies on the effects of specific diets, such as the ketogenic diet, Mediterranean diet, vegetarian diet, and Western diet, on the progression of neuroinflammation and Alzheimer’s and Parkinson’s diseases through the gut-brain axis. The ketogenic diet displays promising potential in ameliorating the clinical trajectory of mild cognitive impairment and Alzheimer’s disease. However, conflicting outcomes were observed among various studies, highlighting the need to consider diverse types of ketogenic diets and their respective effects on clinical outcomes and gut microbiota composition. Vegetarian and Mediterranean diets, known for their anti-inflammatory properties, can be effective against Parkinson’s disease, which is related to inflammation in the gut environment. On the other hand, the westernization of dietary patterns was associated with reduced gut microbial diversity and metabolites, which ultimately contributed to the development of neuroinflammation and cognitive impairment.

**Summary:**

Various studies examining the impact of dietary interventions on the gut-brain axis with regard to neuroinflammation and Alzheimer’s and Parkinson’s diseases are thoroughly reviewed in this article. A strong mechanistic explanation is required to fully understand the complex interactions between various dietary patterns, gut microbiota, and microbial metabolites and the effects these interactions have on cognitive function and the progression of these diseases.

## Introduction

Neurodegenerative diseases cause progressive deterioration in cognitive function by the loss of the activity of neurons in the brain. Worldwide, 10.2 million people suffer from neurodegenerative disorders, and the incidence is steadily increasing. Alzheimer’s disease (AD) is the most common neurodegenerative disease [1]. The World Health Organization has reported that dementia is seen in 50 million people worldwide, with Alzheimer’s patients accounting for 60–70% of these cases. Moreover, this number is envisaged to double every 5 years and will increase to reach 152 million by 2050 [2]. Mild cognitive impairment (MCI) is a state of cognitive impairment that precedes the clinical symptoms of dementia and AD. Therefore, effective intervention in the MCI phase is important to prevent the formation or progression of AD [3]. Parkinson’s disease (PD) is the second most common neurodegenerative disease after AD and is the most common movement disorder worldwide. The prevalence of PD in people over 65 years is 2–3%, and it is estimated that there will be 14.2 million people worldwide to have PD by 2040 [4].

**Fig. 1:**
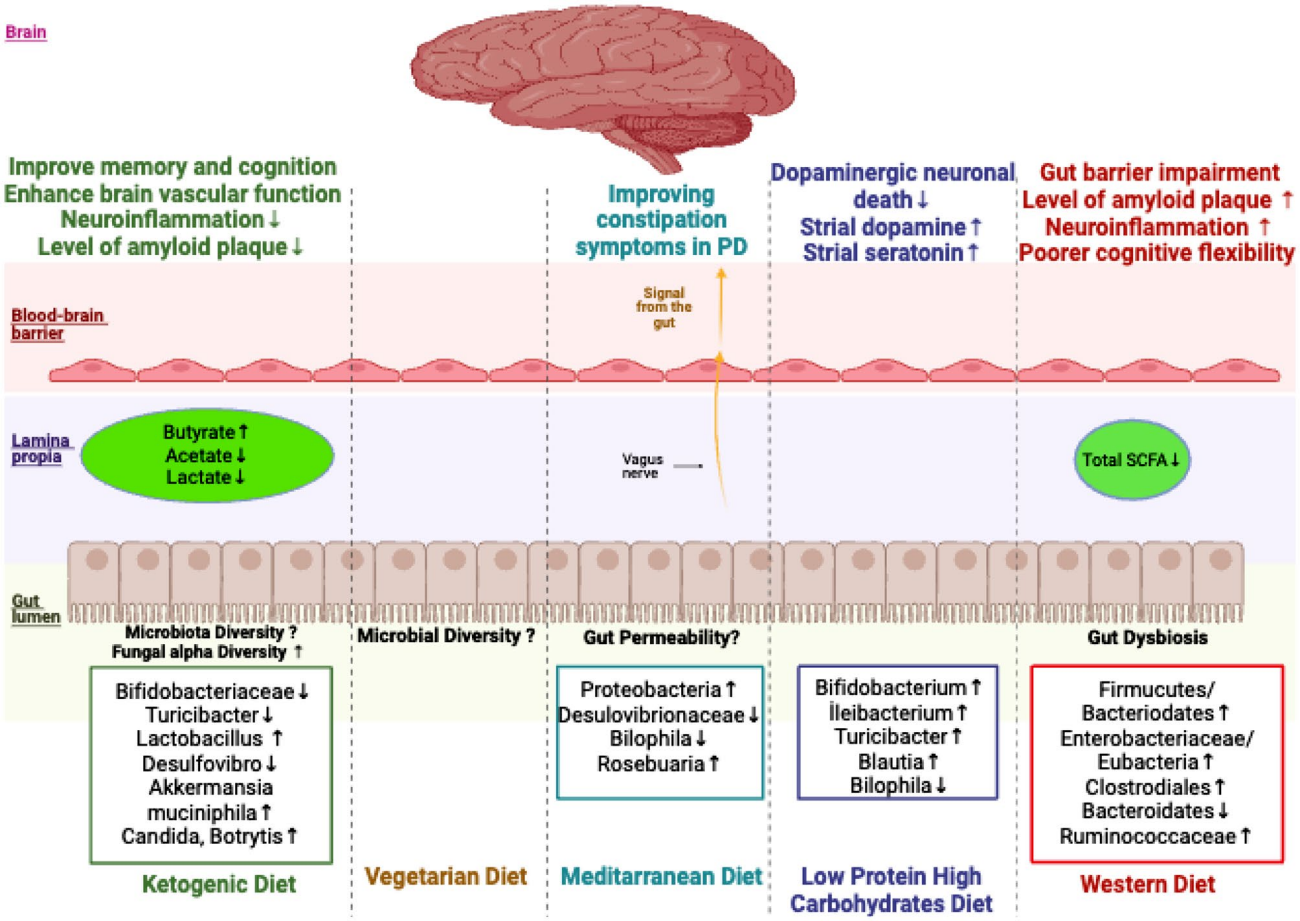
The effects of different types of dietary interventions on the gut-brain axis

Studies have shown that the gut microbiota plays an important role in the pathogenesis of neurodegenerative diseases through the gut-brain axis [5–8]. Recent studies have shown that the gut microbiota is significantly altered in individuals with CI, dementia, and PD [9–11]. The homeostasis of the gut microbiota is impaired with an increase in non-commensal microbes and a decrease in commensal microbes, which is stated as dysbiosis. The signal between the gut-brain axis is also affected by dysbiosis, leading to neurological and neuroimmune abnormalities. Dysbiosis is often associated with deteriorated intestinal epithelium and increased intestinal permeability. Disruption of intestinal epithelial integrity causes systemic inflammation with bacterial translocation and increased levels of interleukin-1 (IL-1), IL-6, and tumor necrosis factor alpha (TNF-α) [12••]. Systemic inflammation leads to neuroinflammation in the blood-brain barrier. Neuroinflammation is the most important factor in the pathogenesis of neurodegenerative diseases. There is a bi-directional interaction between the gut microbiota and the brain. This bi-directional interaction involves many pathways such as immune mechanisms, vagus nerve, and microbial neurometabolite production [13]. This evidence suggests that the gut microbiome is linked to host neurological function via the gut-microbiome-brain axis [14]. Many factors such as genetics, diet, metabolism, age, geography, infection, antibiotic treatment, and stress cause changes in gut microbiota composition and microbial metabolites [15]. Diet is one of the key factors that influence the diversity of the microbiome. Nowadays, it has been discussed that some dietary patterns may have a protective effect against cognitive dysfunction in neurodegenerative diseases by modulation of the gut microbiota [12••, 16••, 17]. The results from four human research studies and eleven animal studies, carefully chosen to investigate the effects of various dietary patterns on the onset and progression of neuroinflammation and Alzheimer’s and Parkinson’s diseases by means of the gut microbiota, are specifically incorporated into this review (Table 1; Fig. 1).

**Table 1:** The effect of dietary intervention on neuroinflammation and Alzheimer’s and Parkinson’s diseases through gut microbiota modulation

**Model**	**Type of ND**	**Followup**	**Neurological evaluation**	**Diet**	**Microbiota change after dietary intervention**	**Comments**	**Study**
**Clinical studies**							
11 MCI 6 CN (Randomized, double-blind, crossover pilot study)	MCI	6 wks MMKD + 6 wks wash out + 6 wks AHAD	Lumbar puncture, CSF biomarkers assays, ApoE e_4 genotyping Aß42, Aß40, total tau, and tau-p181	**MMKD** (< 10% CHO, 60–65% fat, 30–35% prt) **AHAD** (55–65% CHO, 15–20% fat, 20–30% prt)	MMKD induces broader effect on fungal diversity in MCI *Candida*↓ Botrytis↓	MMKD positively modulates the gut microbiome as well as the bacterial metabolites arrays, which in turn may check the overgrowth of opportunistic pathogens such as *Candida*	[3]
11 MCI 6 CN (Randomized, double-blind, crossover pilot study)	MCI	6 wks MMKD + 6 wks wash out + 6 wks AHAD	Lumbar puncture, CSF biomarkers assays, ApoE ε-4 genotyping Aß42, Aß40, total tau, and tau-p181	**MMKD** (CHO < 10%, 60–65% fat, 30–35% prt) **AHAD** (55–65% CHO, 15–20% fat, 20–30% prt)	**-MMKD and AHAD** α- and β-diversity **-MMKD** Enterobacteriaceae↑ *Akkermansia*↑ *Slackia*↑ Christensenellaceae↑ Erysipelotrichaceae↑ *Bifidobacterium*↓ Lachnobacterium↓ -Fecal lactate and acetate↓ -Fecal propionate and butyrate↑	Specific gut microbial signatures may represent the MCI and intervention of MMKD can modulate the gut microbiome and metabolites in association with improved AD biomarkers in CSF	[12••]
11 MCI 6 CN (Randomized, double-blind, crossover pilot study)	MCI	6 wks MMKD + 6 wks wash out + 6 wks AHAD	Metabolites jointly associated with cognitive status	**MMKD** (CHO < 10%, 60–65% fat, 30–35% prt) **AHAD** (55–65% CHO, 15–20% fat, 20–30% prt)	**-MMKD** *Akkermansia muciniphila*↑ *Dialister*↑ *Bacteroides*↑ **-AHAD** *Alistipes* sp. CAG:514↑	AHAD most likely boosted GABA production in MCI patients through a rise in GABA-producing *Alistipes* sp. On the other hand, the GABA-regulating *Akkermansia muciniphila* is more abundant in MCI and CN people following MMKD intervention	[18]
54 PD 16 CN (Case-control study)	PD	14 days	UDPRS III	Ovo-lacto vegetarian diet	No difference	Vegetarian diet containing high anti-inflammatory effects and SCFA can influence on the microbiome and clinical course in patients with PD. However, vegetarian diet takes longer time to change the microbiota and its effect on cognitive function	[16••]
8 PD (dietary intervention) 8 PD (control) Single-arm study	PD	5 wks	MDS-UPDRS MoCA	MD	**MD** α- and β-diversity Proteobacteria↑ Desulfovibrionaceae (phylum Proteobacteria)↓ *Bilophila*↓ *Roseburia* (phylum Firmicutes)↑	A short-term MD intervention could potentially help alleviate constipation symptoms and modulate the gut microbiota in individuals with PD. However, it did not appear to affect intestinal permeability	[19]
**Animal study**							
C57BL/6 male mice (healthy)	Neurovascular function ND	16 wks	Cerebral blood flow BBB function	KD	**Anti-inflammatory** *Akkermansia muciniphila*↑ *Lactobacillus*↑ **Pro-inflammatory taxa** *Desulfovibrio*↓ *Turicibacter*↓	Early KD intervention may improve brain vascular function and metabolic profile, promote beneficial gut microbiota, and these are lower risk for AD	[20]
Male Sprague Dawley rats	AD	8 wks	-Behavioral test *Y*-maze test Water maze test	KD IF HCD	**KD** Proteobacteria↑ **HCD (starch)** Bacteroidetes↑	IF and HCD but not KD may not improve cognitive function, insulin resistance, NI, or the gut microbiome. IF and HCD containing high starch may be beneficial for people with dementia	[21]
C57BL/6 mice	PD	30 days	-Behavioral test Pole test Traction test Rota-rod test Open field test -Striatal dopamine, serotonin, and their metabolites	**LPHC** 77.2% CHO 5% prt 14.4% fat	**LPHC** *Bifidobacterium*↑ *Ileibacterium*↑ *Turicibacter*↑ *Blautia*↑ *Bilophila*↓	This study has shown that the LPHC diet positively changed the gut microbiome and metabolites, raised FGF-21 in the serum and midbrain, protected dopaminergic neurons, and improved motor function in PD mice	[22]
Male C57BL/6 mice (Randomized trial)	Anxiety Memory Cognitive Flexibility	2 wks	-Behavioral test Water maze test Novel object recognition test	**HFD** 42% fat, 43% CHO **HSD** 12% fat, 70% CHO (primarily sucrose)	**HSD** Lactobacillales↑ Clostridiales↑ Bacteroidales↓ **HFD** Erysipelotrichales↑ Clostridiales↑ **HED** Clostridiales↑ Bacteroidales↓	Poorer cognitive flexibility was associated with higher percentages of Clostridiales and lower expression of Bacteroidales in HED. Dysbiosis that is improved by a Western diet may be a factor in cognitive decline	[23]
Male Wistar rats	CI, CD	2, 8, 12, 20, or 40 wks	-Brain immune cell activity -Amyloid-b level -Microglia morphology -Hippocampal reactive oxygen species and apoptosis -Hippocampal synaptic plasticity -Dendritic spine density	SD: 19.77% fat, 51.99% CHO, 28.24% prt) HFD: 59.28% fat, 14.27% CHO, 26.45% prt)	**HFD (2 wks)** Enterobacteriaceae/Eubacteria↑ Serum LPS↑ **HFD (8 wks)** F/B↑	Gut dysbiosis develops in the earliest phase of consumption of an HFD (12 wks), followed by brain pathology, which leads to cognitive decline in obese insulin-resistant rats. Thus, an improvement in gut dysbiosis should provide beneficial effects in the prevention of neuropathology and CD in the obese	[24•]
C57BL/6N mice	NI, neuronal loss	8 wks	-Levels of IL-1β, TNF-α, and IL-6 mRNA -Numbers of Iba-1 + microglia in the hippocampus	High-fructose diet SD	Bacteroidetes↓ Proteobacteria↑	Gut dysbiosis is a critical factor for a high-fructose diet-induced hippocampal NI possibly mediated by impairing the intestinal epithelial barrier	[25]
Male Sprague-Dawley rats	NI and plasticity	2 wks	-Behavioral test Object/location recognition test -mRNA expression of a range of genes involved in inflammation and the neuroplasticity marker -BDNF in the hippocampus and hypothalamus	Control SFA PUFA Sugar	The relative abundance of 89 taxa varied significantly between groups, with the Clostridiales order and its suborders Lachnospiraceae and Ruminococcaceae mostly responsible for these variations	Clostridiales and Lachnospiraceae (genus: unclassified), OTU28 and OTU171, were found to be associated with a HFD and showed a negative correlation with spatial memory	[26]
Male Wistar rats	NI	20 wks (CAF) Omega-3 or water (between 16 and 20 wks)	-Behavioral test Five-trial social memory test	CAF + water CAF + omega 3 SD + water SD + omega 3	**After CAF** Microbial diversity↓ (Chao 1 index) Firmicutes↓ Bacteroidetes↑ Actinobacteria↓ Proteobacteria↑ **After CAF + n3** Microbial diversity (Chao 1 index) Firmicutes↑ Bacteroidetes↓ F/B↑ Actinobacteria	Omega-3 did not show improvements in bacterial diversity; however, it did exhibit a beneficial effect in reducing metabolic endotoxemia caused by the CAF, suggesting its potential advantages in treating neuroinflammation associated with obesity	[27]
Wild-type C57BL/6J male mice	CI	15 wks	-Behavioral test Object location Temporal order memory Nesting behavior tests	FD	Bacteroidetes↓ Proteobacteria↑ Microbial diversity↓ SCFA↓	The FD mice displayed gut microbiota dysbiosis, which was strongly associated with cognitive deficits. Additionally, the FD diet compromised the integrity of the intestinal barrier and led to reduced production of SCFAs	[28]
Male Sprague Dawley rats	NI, CD	8 wks	-Behavioral test Water radial arm maze -Microgliosis	HFD LFD SD	**HFD** Firmicutes↑ Bacteroidetes↓ Actinobacteria↑ Proteobacteria↓	Although a notable shift occurs in the gut microbiome, this alteration does not appear to elicit subsequent impacts on neuroinflammation, as evidenced by the characterization and quantification of microglial cells in the cortex, hippocampus, and hypothalamus	[29]
Triple transgenic (3xtg) mice	CD		-Behavioral test *Y*-maze test -Brain glucose metabolism	HFD SD	Firmicutes↑ Bacteroidetes↓ Rikenellaceae↑ Lachnospiraceae↑ Enterococcaceae↑ S24.7↑ *Clostridium*↑ *Staphylococcus*↑ *Bifidobacterium*↓ *Lactobacillus*↓	A hereditary propensity to neurodegenerative disease and high-fat diets both produce aberrant microbiomes and metabolomes that are cumulative and appear to be detrimental to brain health	[30]
C57Bl6/J mice	CI	22 wks	-Behavioral test *Y*-maze test Novel object recognition test -Hippocampal BDNF	HSPAD	Firmicutes↑ Actinobacteria↑ *Bacteroides*↓ Proteobacteria↓	A prolonged high-saturated-palmitic-acid diet contributes significantly to the development of the obesity phenotype and cognitive impairment by causing gut microbiota dysbiosis, colon inflammation, and circulating LPS	[31]

*BBB* blood–brain barrier, *BDNF* brain-derived neurotrophic factor, *CAF* cafeteria diet, *CD* cognitive decline, *CHO* carbohydrates, *CI* cognitive impairment, *HCD* high-carbohydrate diet, *HFD* high-fat diet, *HSD* high-sucrose diet, *HSPAD* high-saturated-palmitic-acid diet, *FD* fiber deficiency, *F/B* Firmicutes/Bacteroidetes, *LFD* low-fat diet, *LPHC* low-protein high-carbohydrate diet, *MD* Mediterranean diet, *MCI* mild cognitive impairment, *MMKD* Mediterranean modified ketogenic diet, *NI* neuroinflammation, *PD* Parkinson’s diseases, *SCFA* short chain fatty acids, *SD* standard diet, *IF* intermittent fasting

## Methods

**Fig. 2:**
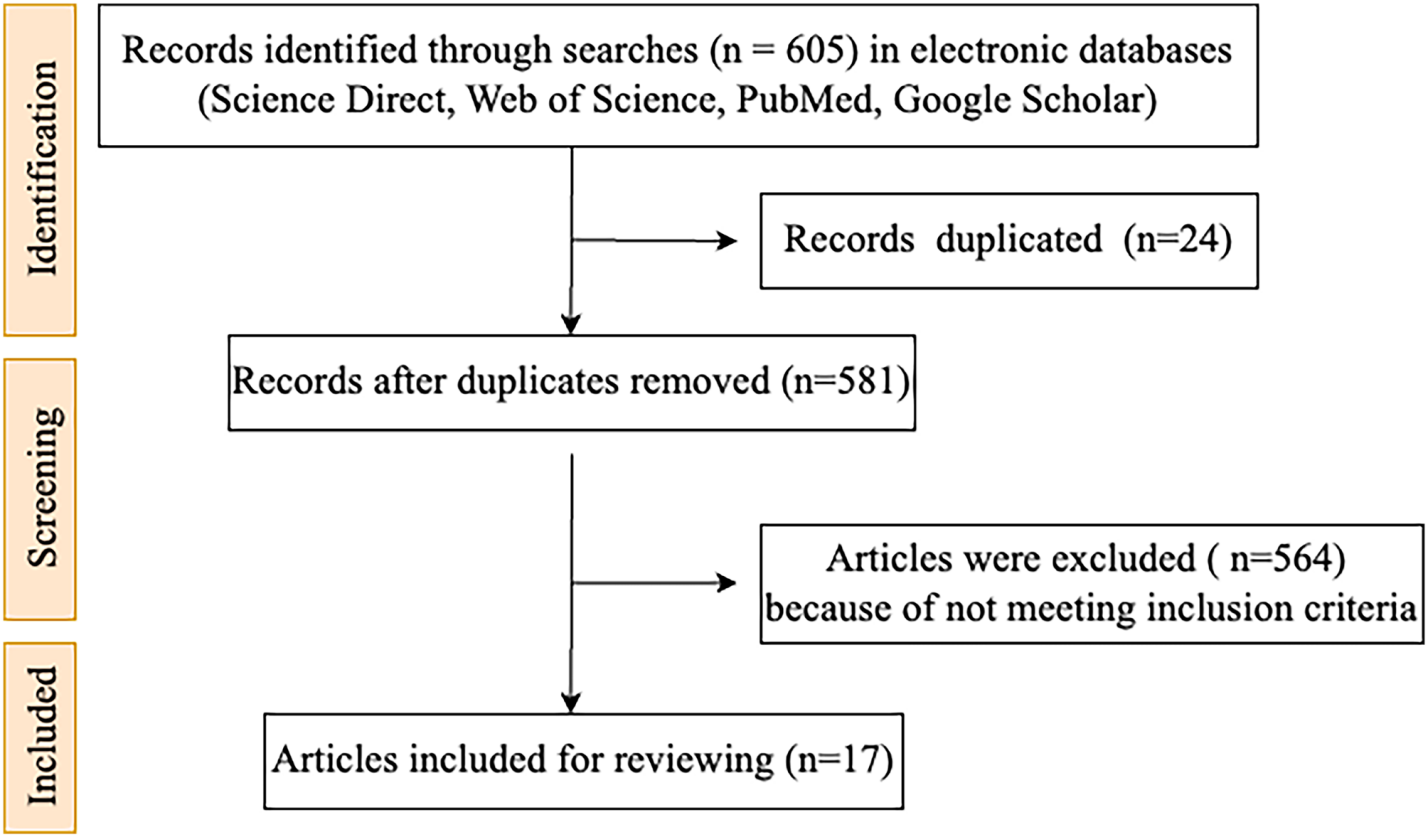
Flow diagram of the literature search and study selection process

Figure 2 illustrates the flowchart of the method used for selecting the research. Literature search was conducted using Web of Science, PubMed, Science Direct, and Google Scholar databases to identify peer-reviewed articles that examined the relationship between dietary intervention, gut microbiota, and neurodegenerative disease and neuroinflammation between 2015 and 2023. For all four databases, the exact search date was from 1 February 2021 through 15 December 2023. A combination of the following keywords was used: “nutrition” or “diet” and “gut microbiota” with “neurodegenerative diseases,” or “neuroinflammation” or “cognitive decline” or “alzheimer’s disease” or “parkinson’s disease.” This review considers only studies published between 2015 and 2023 and excludes those in languages other than English in this review. Additionally, short communication letters, conference abstracts, book chapters, short surveys, and studies based solely on authors’ perspectives were excluded. The review comprises 17 selected studies investigating the connection between diet patterns and their impact on gut microbiota concerning cognitive impairment, cognitive decline, neuroinflammation, Alzheimer’s, and Parkinson’s diseases (Table 1). The studies were selected by two researchers (ŞA, SB) by checking first the titles and abstracts and then the full-text articles.

## Results

### Ketogenic Diet, Gut Microbiota, and Alzheimer’s Disease

Pathology of Alzheimer’s disease (AD) is characterized by extracellular accumulations of amyloid-β-peptides (Aβ) in the senile plaques and by intracellular depositions of hyper-phosphorylated tau proteins that form neurofibrillary tangles [32]. Studies have shown that brain amyloid plaque accumulation is the result of years of chronic inflammation in the central nervous system (CNS) and the periphery. It is stated that brain amyloid deposition begins 10–20 years before clinical indications. Mild cognitive impairment (MCI) is a transitional stage between normal cognitive aging and dementia, particularly AD. It is a critical period for early detection and intervention as it offers an opportunity to prevent or delay the onset of dementia. Therefore, intervening during the MCI phase is crucial for preserving cognitive function and maintaining the quality of life of affected individuals [33, 34]. However, the pathogenesis of the disease is still not clear. Despite ongoing research efforts, no definitive treatment method has been discovered to prevent or slow the progression of these diseases.

In recent years, the gut microbiota is considered an additional organ of our body, defined as “the second brain.” In the past few years, the direct causal effects of gut microbiome on the neuronal system and the brain have been shown [35]. Recent studies have put forward that gut dysbiosis may affect the synthesis and secretion of several brain-derived neurotrophic factors and gut microbial-derived metabolites widely associated with cognitive decline and dementia [36, 37]. Therefore, modulation of the gut microbiome may induce positive effects on neuronal pathways that can decelerate the progression of AD [38]. Emerging evidence suggests that specific dietary patterns and nutrients affect gut microbiota composition which is associated with the production or aggregation of amyloid proteins [39, 40]. In turn, some dietary patterns that modulate gut microbiota may be an effective method to prevent the development of AD and dementia.

The first studies showing that the ketogenic diet (KD) that provides fasting or fasting mimicking may have a beneficial effect on neurodegenerative diseases were published at the beginning of the twentieth century. The KD is a type of high-fat and low-carbohydrate diet and is firstly and generally used in the treatment of drug-resistant epilepsy [41] and also is highly effective in the treatment of resistant epilepsy. Over time, its application in other neurodegenerative disease was studied including amyotrophic lateral sclerosis [42], traumatic brain injury [43], cerebral ischemia [44], and neurodegenerative diseases (Parkinson’s and Alzheimer’s diseases) [45]. The KD approach has been proposed as a potential therapeutic strategy for AD, based on targeting the underlying pathophysiological mechanisms of the disease. Impaired glucose metabolism is common in AD. Decreased glucose uptake in the brain plays a role in the development of AD neuropathology. KD alters metabolic pathways from glucose whose metabolic pathway is toxic to fatty acids and ketone bodies. Thus, the brain is protected from inflammatory and toxic effects that impaired glucose metabolism causes. Moreover, the KD has numerous potential targets, which may contribute to its anti-inflammatory effects, including adenosine, ketone bodies, mTOR pathways, PPARγ, NLRP3 inflammation, and the modulation of gut microbiota [20]. However, further research is needed to fully understand the mechanisms underlying the anti-inflammatory effects of the KD including gut microbiota on AD and to identify other potential targets for intervention.

After reviewing studies in the literature, five of them were found to explore the impact of a ketogenic diet on gut microbiota and its connection to AD and cognitive impairment (CI). A summary of their findings is presented in Table 1. In 2018, Ma et al. in an animal study showed that 16 weeks of KD increased the relative abundance of putatively beneficial gut microbiota (*Akkermansia muciniphila* and *Lactobacillus*) and reduced that of putatively pro-inflammatory taxa (*Desulfovibrio* and *Turicibacter*). Thus, they found that the KD intervention in the early stage, which increases beneficial gut microbial taxa, may enhance brain vascular function, improve metabolic profile, and reduce the risk for AD [20].

Three randomized, double-blind, crossover pilot studies were conducted to compare the effects of a modified Mediterranean ketogenic diet (MMKD) and an American Heart Association Diet (AHAD) intervention in older adults with MCI versus those who were cognitively normal (CN). Each study included a 6-week intervention period of either MMKD or AHAD, followed by a 6-week washout period, and then another 6-week intervention period of the other intervention. While one of them has investigated the effect of MMKD on bacterial microbiomes [12••], the other has been related to fungal microbiomes [3]. The most recent one specifically used data sparsity and compositionality to evaluate the relationships between these diets and cognitive status and the microbiome, foodome, and metabolome [18].

Maintaining a classic KD can be challenging over the long term, which may limit its effectiveness for patients with AD. However, adopting less strict ketogenic diets, such as the modified Atkins diet, could help improve compliance with the dietary regimen. These diets provide greater flexibility and palatability, which can increase adherence to the less strict KD type. MMKD has been suggested as related to CI in the literature. MMKD, which mainly points to olive oil and fish as healthy sources of fats and proteins, allows for slightly higher carbohydrate consumption to provide an increased intake of vegetables and fruits. Nagpal et al. show that the MMKD alters the gut microbiome signature and short-chain fatty acids (SCFAs), and these changes are associated with improved cerebrospinal fluid (CSF) AD biomarkers in older adults. While there were no significant differences in the α- and β-diversity of the gut microbiome between groups, it has been observed that both MMKD and AHAD interventions led to variations in the levels of specific gut bacteria between the MCI and CN (cognitively normal) subjects. The phylum Actinobacteria, family Bifidobacteriaceae, and genus *Bifidobacterium* are significantly decreased in MCI group after MMKD as compared to CN counterparts and AHAD group participants. The study also revealed that the MMKD may lead to a slight increase in the production of beneficial SCFAs, including butyrate. The increased level of butyrate following the MMKD may have a positive impact on reducing gut leakiness and limiting the diffusion of lipopolysaccharides (LPS), thus promoting better brain health. Additionally, the observed decrease in acetate and lactate levels in the MCI-MMKD group could be linked to improved memory and cognitive function in MCI participants [12••].

Furthermore, Nagpal et al. conducted the first-ever study to investigate the fungal microbiome in older adults with MCI in comparison to age-matched counterparts with CN. They found that the MMKD induced an increase in fungal alpha-diversity in patients with MCI but not in their CN counterparts. This result is remarkable because the alpha-diversity was lower in patients with MCI at the baseline and was increased (restored) by MMKD in these patients. The genus-level mycobiome composition is also modulated more heavily and somewhat positively by MMKD than AHAD intervention. The abundance of *Botrytis*, which was higher in MCI group at the baseline, has been decreased by MMKD in MCI but not in CN subjects. Also, the significant reduction has been seen in the proportion of genus *Candida* in the MCI-MMKD group. The *Candida* genus encompasses numerous opportunistic species that have been associated with various gut-related disorders, such as inflammatory bowel diseases and gut inflammation. Notably, they found a significantly negative correlation of *Candida* with short-chain fatty acid butyrate [3], thus hinting toward a positive effect of MMKD on fungal flora, particularly in the MCI group. These findings have indicated that the intervention of MMKD may modulate the gut microbiota at the fungal and bacterial levels and as a result improve AD biomarkers in patients with AD or CI. A recent study has shown that there was a higher relative abundance of Akkermansia bacteria in the MMKD group compared to the AHAD group. Also, there was an increase in *Dialister* and *Bacteroides* bacteria in the CN group compared to the MCI group. Specifically, they found various types of *Akkermansia muciniphila* were more prevalent in the MMKD group but not in the AHAD group. Among CN in the MMKD group, there was an increase in *Dialister invisus* and several strains of *Bacteroides fragilis*. The ratio of Alistipes bacteria to *Bifidobacterium adolescentis* was significantly different between the MCI and CN groups, but this change occurred after the AHAD, not the MMKD. This effect was observed only in the MCI group, indicating that people at different cognitive levels may respond differently to interventions that affect the microbiome. Additionally, the authors found that individuals carrying the APOE4 gene had more microbes capable of producing GABA, a neurotransmitter, and consequently more GABA in their CSF. They proposed that MMKD could help people with MCI by modifying gut-transit time and GABA levels [18].

On the other hand, Park et al. investigated how ketone-producing regimens affected CI, inflammation, glucose metabolism, and gut microbiota. Following an injection of amyloid-ß into the hippocampus, rats underwent either an 8-week ketogenic diet (AD-KD), intermittent fasting (AD-IMF), a diet containing 30% fat (AD-CON), or a high carbohydrate (starch) diet (AD-CHO). By raising Proteobacteria, AD-KD made gut dysbiosis worse, while AD-CHO made it better by raising Bacteroidetes. Additionally, they have shown that AD-CON, AD-IMF, and AD-CHO, but not AD-KD, reduced hippocampus amyloid-ß deposition and enhanced memory performance [21].

Individuals with AD often exhibit impaired glucose uptake in the brain, but their ability to utilize ketones remains intact. While some studies suggest potential benefits of KD on CI and AD [3, 12••], others indicate possible limitations or even adverse effects of it. Types of fats utilized in the ketogenic diet may play a crucial role in determining metabolic outcomes and their impact on cognitive function. Furthermore, the effect of different types of ketogenic diets on gut microbiota is still far from conclusive. Moreover, some side effects of KD such as loss of body weight, deterioration in lipid absorption, and its low sustainability are considered disadvantages [46]. Future research efforts should aim to explore these connections more comprehensively, considering the potential interactions between specific components of different types of KD, gut microbiota, and cognitive function.

### Vegetarian, Mediterranean, and Low-Protein High-Carbohydrate Diets, Gut Microbiota, and Parkinson’s Disease

Parkinson’s disease (PD) is the second most common neurodegenerative disorder worldwide, characterized by key motor symptoms like resting tremor, bradykinesia, rigidity, and postural instability [47]. It is linked to the abnormal build-up of α-synuclein protein, leading to the loss of dopamine-producing neurons in the substantia nigra [48]. Recent research suggests that early PD-related changes, such as constipation and anosmia, align with histological findings in the gut, including the presence of Lewy bodies. Constipation is a prevalent symptom in PD, impacting around 61.4% of patients. Remarkably, approximately 24.5% of these individuals reported experiencing bowel discomfort prior to the onset of the characteristic motor symptoms associated with PD [49]. This connection supports the idea that PD may have its origins in gut inflammation, possibly mediated through the gut-brain axis [50]. Studies indicate a higher occurrence of PD in individuals with chronic inflammatory bowel disease (IBD), and markers of immune system activity like calprotectin are elevated in stool samples of PD patients [51]. Impaired intestinal barrier function and microbial imbalances in the gut may contribute to the translocation of pro-inflammatory cytokines and endotoxins into the colon, which can promote systemic inflammation, ultimately impacting the misfolding of α-synuclein and exacerbating PD pathology [52].

Recent studies have highlighted the significant role of the gut microbiota in PD pathogenesis. Specific bacteria, more prevalent in the guts of individuals with PD, produce endotoxins that trigger inflammation and promote methane production [53]. Certain gram-negative Enterobacteriaceae strains, like *Escherichia coli* and *Salmonella*, were found to secrete pro-inflammatory lipopolysaccharides (LPS) linked to motor symptom severity [54]. Patients with PD have higher levels of methane-producing bacteria such as *Christensenella* spp. and *Methanobrevibacter*, which increases intraluminal pressure and reduces peristaltic movements, resulting in constipation [55]. Conversely, decreased anaerobic bacteria, such as Prevotellaceae, involved in anti-inflammatory pathways, were found in PD patients. The Prevotellaceae family is one of the mucin-producing commensals that secrete SCFAs from the fermentation of dietary fibers such as butyrate. Decreased populations of butyrate-producing anaerobic microbes like Roseburia and Faecalibacterium were observed in this patient group. Reduced butyrate production is associated with elevated oxygen levels in the gut mucosa, which increases the risk of oxidative damage [56].

In the context of gut inflammation in PD, researchers have investigated vegetarian diets rich in SCFA due to their anti-inflammatory properties. A study by Hegelmaier et al. demonstrated that a vegetarian diet positively influenced the microbiome composition and clinical trajectory of PD patients (Table 1). The diet reduced Proteobacteria and Firmicutes associated with gut dysbiosis and inflammation. Notably, the Unified Parkinson Disease Ratings Scale (UPDRS III) improved, and levodopa-equivalent daily doses decreased after a 1-year follow-up with a vegetarian diet and fecal enema. A negative correlation was found between the Shannon index and UPDRS III. The abundance of Ruminococcaceae was associated with UPDRS III, and enema reduced Clostridiaceae abundance. However, beta-diversity remained unchanged after the intervention, possibly due to the small sample size and short duration of the dietary intervention [16••].

Since the 1970s, several studies have shown the positive outcomes of protein-restricted diets in fluctuating Parkinsonians due to a significant interaction between dietary proteins and patients’ responses to levodopa [57]. Chu et al. investigated whether the gut microbiota and metabolites are involved in the LPHC diet’s potential to improve movement disorders in 1-methyl-4-phenyl-1,2,3,6-tetrathydropyridine (MPTP)-induced PD in mice [22]. The increased abundance of the genera *Bifidobacterium*, *Ileibacterium*, *Turicibacter*, and *Blautia* and the decreased abundance of the genera *Bilophila*, *Alistipes*, and *Bacteroides* showed that the LPHC diet restored the imbalance in the composition of gut bacteria in PD mice. Metabolomic analyses in the serum and feces have shown that the LPHC diet significantly raised the level of aromatic amino acids (AAAs), such as tryptophan, tyrosine, and phenylalanine. Elevated AAA concentrations in the gut may result from an LPHC diet’s higher carbohydrate availability in the large intestine and reduced microbial degradation of AAA. The circulation’s AAA levels rise as a result of gut AAAs crossing the gut epithelium and entering the bloodstream, which also elevates dopamine and 5-HT levels in the central nervous system. Also, they have demonstrated that the LPHC diet elevates serum concentrations of bile acids, including TUDCA and taurine, whose neuroprotective effects are well studied [58, 59]. Ultimately, the LPHC diet attenuated movement deficits in MPTP-induced PD. AAAs, microbial metabolites (taurine and TUDCA), and FGF-21 were identified as important mediators along the gut-microbiota-brain axis for this role of the LPCH diet on PD [22].

A human study assessed the feasibility of a Mediterranean diet intervention and its impact on gut microbiota composition, intestinal permeability, and gastrointestinal function in PD patients. The study has shown that after 5 weeks of dietary intervention, constipation syndrome scores went down. *Bilophila*, which was initially higher in PD, slightly decreased after the dietary intervention. Notably, the proportion of *Roseburia* was significantly lower in PD compared to controls at the beginning and increased after intervention. However, there were no differences in markers of intestinal permeability between the control and PD groups, both before and after the intervention [19]. MD can be feasible to attenuate GI symptoms in PD through gut microbiota modification.

In summary, these findings emphasize the crucial role of gut microbiota and metabolites in PD and suggest that dietary interventions, such as vegetarian, Mediterranean, and low-protein diets, may offer potential benefits in alleviating inflammation and GI symptoms and improving clinical outcomes in PD patients through the gut-brain axis.

### Western Diet, Gut Microbiota, and Neuroinflammation

**Fig. 3:**
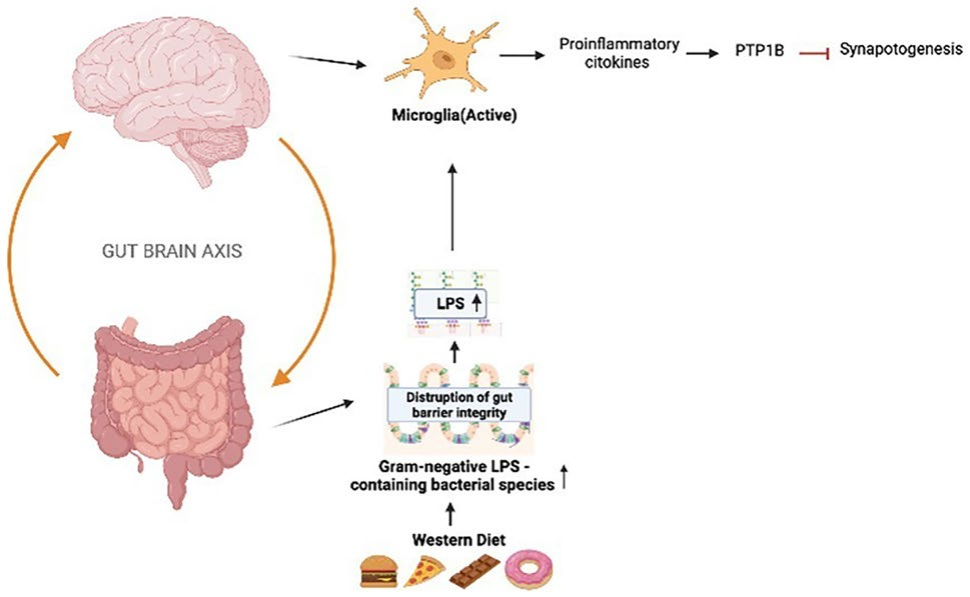
Mechanistic explanation of the relationship between Western diet, gut microbiota, and neuroinflammation

The Western diet, characterized by high levels of fat, animal proteins, and refined carbohydrates, is associated with the onset of neuroinflammation, which plays a role in neurodegenerative diseases. In recent times, the impact of the Western diet on cognitive function and gut health has become a matter of increasing concern. The Western-style diet disrupts the balance of gut microbiota, leading to intestinal barrier damage and increased permeability. This disruption results in bacterial endotoxins like LPS to enter the bloodstream [31]. LPS is primarily produced by gut gram-negative bacteria and has been linked to diseases like AD. Studies show positive correlations between AD and the presence of gram-negative bacteria such as *Helicobacter pylori*, *Porphyromonas gingivalis*, *Prevotella melaninogenica*, and *Campylobacter rectus* [60]. When LPS enters the bloodstream, it can trigger a response involving TLR4-dependent CD14 upregulation in intestinal cells, further damaging the intestinal barrier and increasing gut permeability. This process activates microglia, the primary immune cells in the central nervous system, in the hippocampus, leading to increased pro-inflammatory cytokine expression [61]. These cytokines stimulate the transcription of protein tyrosine phosphatase 1B (PTP1B), which inhibits critical signaling pathways required for synaptic function (pIRS-pAKT-pGSK3β). PTP1B causes synaptic disruption and neuroinflammation [62] (Fig. 3). Neuroinflammation plays a pivotal role in the pathogenesis of neurodegenerative disease. Seven distinct studies have been evaluated in the field of understanding the complex interaction between the Western diet, gut microbiota, and the beginning or progression of neurodegenerative diseases (Table 1). Most of them focused on a high-fat diet particularly a high saturated-fat diet, which is mainly characterized by a Western diet.

Saiyasit et al. found that a high-fat diet (HFD) caused changes in the composition of intestinal bacteria in rats. Specifically, the ratio of Enterobacteriaceae to Eubacteria increased in the second week of HFD, and the Firmicutes/Bacteroidetes (F/B) ratio increased in the eighth week. These changes in the gut microbiota were associated with gastrointestinal and systemic inflammation. Furthermore, their study demonstrated that prolonged consumption of HFD for 40 weeks led to an increase in amyloid plaque levels in the brain. The mechanism behind this increased amyloid production in the brain with prolonged HFD consumption is attributed to bacterial amyloid production resulting from intestinal dysbiosis. It has been observed that intestinal dysbiosis leads to elevated levels of bacterial amyloid in the peripheral nervous system, as bacterial amyloid can cross the blood-brain barrier similar to circulating prions. The increased amyloid in the brain triggers protein misfolding, thereby promoting neuroinflammation. As a result, prolonged HFD consumption not only induces cognitive dysfunction but also contributes to the progression of AD, accompanied by elevated brain amyloid levels. These findings strongly support the “gut-brain” hypothesis, suggesting that gut dysbiosis plays a role in the early stages of dementia development, even before CI becomes apparent [24•].

Magnusson et al. conducted an animal study comparing the effects of HDF and a high-sucrose diet (HSD) on the microbiome and cognitive function. They found that both diets resulted in alterations in the microbiome compared to a normal diet, with some similarities between the two (e.g., increased Clostridiales). However, the HSD showed a greater decrease in Bacteroidetes compared to the HFD. Interestingly, Lactobacillales were significantly increased only in the HSD group, while Erysipelotrichales were significantly affected only by the HFD. In terms of cognitive function, the researchers observed that higher percentages of Clostridiales and lower expression of Bacteroidales in the microbiome of mice on high-energy diets were associated with poorer cognitive flexibility during the reversal trials. These findings suggest that changes in the microbiome may play a role in the cognitive changes associated with consuming a Western diet. The specific alterations in bacterial groups related to the type of diet highlight the potential influence of different dietary components on gut microbiota composition and its impact on cognitive flexibility [23].

High fructose, the main refined carbohydrate source of the Western diet, causes the hippocampal neuroinflammatory response and neuronal loss, but the underlying mechanisms remained elusive. Lie et al. examined alterations in the gut microbiota and intestinal epithelial barrier as the causes of hippocampal neuroinflammation induced by a high-fructose diet in mice. Gut microbiota compositional alteration, SCFA reduction, intestinal epithelial barrier impairment, NOD-like receptor family pyrin domain-containing 6 (NLRP6) inflammasome dysfunction, high levels of serum endotoxin, and FITC-dextran were observed in fructose-fed mice. This study showed that gut dysbiosis is a critical factor for a high-fructose diet-induced hippocampal neuroinflammation in C57BL/6N mice possibly mediated by impairing intestinal epithelial barrier. The faulty colonic NLRP6 inflammasome is responsible for intestinal epithelial barrier impairment. SCFAs can stimulate NLRP6 inflammasome and ameliorate the impairment of the intestinal epithelial barrier, resulting in the protection against a high-fructose diet-induced hippocampal neuroinflammation and neuronal loss. These results provide new evidence for the protective mechanisms of SCFAs and pioglitazone against hippocampal neuroinflammatory response and neuronal loss in this animal model [25].

In their study, Beilharz et al. looked at the effects of various macronutrients on markers for memory, neuroinflammation, and neuroplasticity, as well as the gut microbiota. For 2 weeks, identical pure diets of control, sugar, saturated fatty acid (SFA), or polyunsaturated fatty acid (PUFA) were given to rats. Importantly, each diet altered the microbial composition differently. Specifically, the relative abundance of 89 taxa differed significantly between groups with the majority of these changes accounted for by the Clostridiales order and within that Lachnospiraceae and Ruminococcaceae. These taxa showed a range of macronutrient-specific correlations with place memory. At the species level, no significant relationship was found between microbial abundance and spatial memory. However, at the operational taxonomic unit (OTU) level, spatial memory showed a negative correlation with *Oscillibacter*_OTU30 (*r* = 0.39, *p* = 0.047) and a positive correlation with *Barnesiella*_OTU114 (*r* = 0.43, *p* = 0.019). It was determined that *Oscillibacter* shows a negative correlation with spatial recognition memory. *Oscillibacter* is known to increase in obesity and is associated with gut permeability. Therefore, the SFA group may have increased gut permeability, leading to higher endotoxin plasma levels and more peripheral inflammation. Chronic exposure to a high-saturated fat diet can damage the blood-brain barrier (BBB), resulting in increased neuroinflammation through the leakage of peripheral proteins from the blood to the brain. Clostridiales and Lachnospiraceae (genus: unclassified), OTU28 and OTU171, were found to be associated with a HFD and showed a negative correlation with spatial memory. On the other hand, OTU40 and OTU99 were associated with a high-CHO diet and showed positive and negative correlations with spatial memory, respectively. Recent studies investigating the effects of high-energy diets on microbial composition have shown that the OTU analysis level is crucial, especially for determining microbial signatures following short-term dietary interventions [26]. Another study has investigated gut microbiota-metabolomics signatures preceding dementia in genetically prone (3xtg) mice, with and without a high-fat diet intervention. Analysis of the metabolites showed that 3xtg mice, especially when on a high-fat diet, had lower levels of unsaturated fatty acids and choline and higher levels of ketone bodies, lactate, amino acids, trimethylamine, and trimethylamine N-oxide. These changes in metabolism were associated with a higher presence of Enterococcaceae, *Staphylococcus*, *Roseburia*, *Coprobacillus*, and *Dorea* bacteria and a lower presence of S24.7, rc4.4, and *Bifidobacterium*. These bacterial changes were linked to cognitive decline and reduced brain metabolism [30].

The Western diet is characterized by a fiber-deficient diet. In a study, they investigated the effects of chronic dietary fiber deficiency (FD) on cognition using a mouse model. The FD mice displayed gut microbiota dysbiosis, specifically decreased Bacteroidetes and increased Proteobacteria, which was strongly associated with cognitive deficits. Notably, a rapid shift in gut microbiota composition was observed in mice subjected to a short-term FD diet (7 days) before experiencing cognitive impairment, indicating a potential causal relationship between gut microbiota profiles and cognitive outcomes. Additionally, the FD diet compromised the integrity of the intestinal barrier and led to reduced production of SCFAs. These findings shed light on the critical role of dietary fiber in maintaining gut microbiota balance and its implications for cognitive health [28].

A recent study assessed the effects of omega-3 (n3) supplementation on various aspects including intestinal microbiota, fatty acid profiles, neuroinflammation, and social memory in rats fed a cafeteria diet (CAF). Male Wistar rats were fed with CAF for 20 weeks. Omega-3 (500 mg/kg/day) was supplemented between the 16th and 20th weeks. CAF reduced microbiota diversity, while omega-3 did not show improvements in bacterial diversity; however, it did exhibit a beneficial effect in reducing metabolic endotoxemia caused by the CAF, suggesting its potential advantages in treating neuroinflammation associated with obesity. In the social memory test, CAF-fed animals showed greater social interaction with no effect of n3. However, they stated that this result could not affirm that the dysbiosis caused by CAF was the cause of the increased sociability [27].

A study hypothesized that a high-fat diet would result in dysbiosis of the gut microbes, which would then lead to neuroinflammation and cognitive loss. This study investigated the effects of an 8-week high-fat diet on water radial arm maze performance, gut bacteria diversity and abundance, and microgliosis in male Sprague Dawley rats who were 7 months old. In contrast to a low-fat, control diet, they discovered that a high-fat diet changed the populations of gut microbes. However, neither microgliosis nor maze performance—a test of spatial working memory—showed significant differences across dietary groups [29].

A study investigated whether changes in the gut microbiota, colon inflammation, and cognition are driven by the high-saturated-PA diet which is the main saturated fatty acid of the Western diet. The diversity and richness of the gut microbiota decreased significantly in the diet-induced obese mice. At the phylum level, they found that the gut microbiota had increased Firmicutes and Actinobacteria and decreased *Bacteroides* and Proteobacteria. Decreased Bacteroidetes were linked to poor recognition memory and spatial memory. Additionally, mice showed decreased tight junction proteins, elevated plasma LPS, and increased colon and liver inflammation. The hippocampal brain-derived neurotrophic factor decreased significantly [31].

## Conclusion

In conclusion, maintaining a healthy balance between the host and gut microbiota is essential for optimal health and disease prevention, particularly in the context of neurodegenerative diseases and neuroinflammation. Diet plays a significant role in shaping the composition of the gut microbiota, and dietary interventions have the potential to modulate the gut microbiota and promote neuroprotection. Due to a lack of conclusive human trial data that definitively proves a causal relationship between a particular diet and microbially mediated brain functions and symptoms, there are still gaps in our knowledge to decipher these complex relationships. There are limited studies in the field in the literature, which are mainly animal studies. Based on restricted knowledge, a ketogenic diet (KD) can be effective, especially at the cognitive impairment stage, through gut microbiota modulation. However, the type of KD is a key factor in evaluating the clinical outcomes of cognitive impairment and Alzheimer’s disease related to gut microbiota and metabolites. Parkinson’s disease (PD), which is closely linked to the inflammatory environment in the gut, can be managed with a vegetarian and Mediterranean diet via the gut-brain axis. Also, a low-protein high-carbohydrate diet, one of the effective dietary interventions on PD, has been shown to improve disease progression by modulating gut microbiota. Western dietary patterns can change the gut microbiota in ways that cause neuroinflammation and eventually result in the onset of cognitive decline. Overall, there is a need for a robust mechanistic explanation of the intricate interplay between different types of dietary patterns, gut microbiota, microbial metabolites, and their consequences on cognitive function and neurodegenerative disease progression. 
